# Untargeted Metabolomics Reveals Metabolic Reprogramming During Viable but Non-Culturable State Formation in *Aeromonas hydrophila* Under Preservative Stress

**DOI:** 10.3390/foods15081289

**Published:** 2026-04-09

**Authors:** Gururaj Moorthy, Jatuphol Pholtaisong, Anusara Wongkotsila, Soottawat Benjakul, Awanwee Petchkongkaew, Jirakrit Saetang

**Affiliations:** 1 International Center of Excellence in Seafood Science and Innovation, Faculty of Agro-Industry, Prince of Songkla University, Hat Yai 90110, Songkhla, Thailand; 6611030010@email.psu.ac.th (G.M.); soottawat.b@psu.ac.th (S.B.); 2 Department of Biotechnology, Faculty of Science and Technology, Thammasat University (Rangsit Campus), Khong Luang 12120, Pathum Thani, Thailand; 3 Thammasat University Center of Excellence in Global Food Security, Thammasat University (Rangsit Campus), Khong Luang 12120, Pathum Thani, Thailand; 4 Department of Pathology, Faculty of Medicine, Prince of Songkla University, Hat Yai 90110, Songkhla, Thailand; 5 International Joint Research Centre for Food Security, School of Food Science and Technology, Faculty of Science and Technology, Thammasat University (Rangsit Campus), Khong Luang 12120, Pathum Thani, Thailand

**Keywords:** *Aeromonas hydrophila*, VBNC, food safety, preservatives, metabolome

## Abstract

*Aeromonas hydrophila* is a major seafood-borne pathogen capable of persisting under preservative-associated stress by entering a viable but non-culturable (VBNC) state, thereby evading culture-based detection. Here, untargeted metabolomics was applied as the primary analytical approach to elucidate metabolic reprogramming during VBNC formation under seafood-relevant preservation conditions. Cells were incubated at 4 °C for 30 days in sodium benzoate-supplemented saline, comparing 0.85% NaCl (culturable condition) and 4% NaCl (VBNC-inducing condition), with sampling every 6 days. Under 4% NaCl with sodium benzoate, culturability declined from 6.18 log CFU/mL at day 0 to undetectable levels by day 30, while cell viability was retained, confirming VBNC induction. UHPLC–ESI–QTOF–MS profiling detected over 893 intracellular metabolic features, of which 518 metabolites were significantly altered between VBNC and culturable states at day 30. Principal component analysis revealed clear, time-dependent metabolic divergence, with the VBNC trajectory explaining 34.4% (PC1) and 11.5% (PC2) of total variance. Pathway enrichment analysis demonstrated significant remodeling of alanine, aspartate and glutamate metabolism (8/28 hits, FDR = 5.7 × 10^−4^); arginine biosynthesis (5/14 hits, FDR = 5.44 × 10^−3^); purine metabolism (10/70 hits, FDR = 8.34 × 10^−3^); and pyrimidine metabolism (7/39 hits, FDR = 1.35 × 10^−2^), indicating nitrogen conservation and metabolic downshifting. A robust biomarker panel, including depleted cyclic AMP, aminoadipic acid, hypotaurine, O6-CM-dG, and betaine, and enriched urocanic acid, pipecolic acid, proline, azelaic acid, and orcinol perfectly discriminated VBNC from culturable cells. These findings demonstrate that sodium benzoate-based preservation can induce a metabolically reprogrammed VBNC state in *A. hydrophila*, highlighting a hidden food safety risk beyond culture-based assessment.

## 1. Introduction

Seafood possesses global economic importance and popularity among consumers, as evidenced by its trade volume surpassing that of other meat products [1]. Consequently, guaranteeing seafood safety is not merely a public health concern but also of paramount importance in the seafood industry and a vital economic element that needs to be stabilized. The perishable nature of seafood products and foodborne disease outbreaks caused by pathogenic microorganisms has made strict food safety regulations a mandatory one. An emerging concern is that despite the implementation of rigorous disinfection methods and effective food safety protocols, pathogenic microorganisms frequently surpass these safeguards, resulting in outbreaks of various foodborne diseases [2].

A significant challenge in foodborne pathogen management is the existence of viable but non-culturable (VBNC) microorganisms. The VBNC state is characterized as a self-protective and survival tactic performed by many bacteria when it is exposed to adverse and unfavorable environmental conditions [3]. Interestingly, these bacteria exhibit extraordinary tolerance to adverse conditions, lower metabolic activity and preserve viability markers, thereby retaining their virulent potential while being undetectable and unquantifiable using standard culture-based methods [4]. This unculturability of these pathogens makes the conventional microbial detection procedures ineffective, posing a substantial threat to the food industry and public health [5]. The physiological modification of the VBNC state can be stated as an adaptive survival mechanism which enhances long-term survivability in adverse settings from which these cells may eventually recover later and regain culturability and pathogenicity, if conditions become favorable again [6].

*A. hydrophila*, a Gram-negative facultative anaerobic pathogen belonging to the Aeromonadaceae family, is the most common foodborne pathogen and spoilage bacteria globally [7]. Ubiquitous in aquatic environments and various seafood products, *A. hydrophila* can cause human gastroenteritis with symptoms including nausea, diarrhea and abdominal pain [8]. In terms of pathogenicity, incidence of acute diarrhea associated with the contamination of cold dishes and shrimp salad and its prevalence as a seafood-borne pathogen has been well documented in multiple countries in seafood like mussels, oysters, and clams, thereby underscoring its importance as a notable foodborne pathogen [9,10]. The presence of multi-drug-resistant *A. hydrophila* strains in seafood poses a significant food safety concern, as these resistant variants can be transmitted to humans by consumption of contaminated seafood products [11]. Furthermore, *A. hydrophila* is a psychrotrophic bacterium, capable of growing at temperatures as low as 4 and even −1 °C, complicating its management in refrigerated processed seafood products as it prone to exhibiting pathogenicity and causing foodborne outbreaks [12,13]. In addition, the bacterium is recognized for expressing virulence factors under adverse conditions, employing both lateral and polar flagella for locomotion and forming biofilms, all of which enhance its persistence and antimicrobial resistance [8]. Studies have shown that *Aeromonas* species have shown tolerance to nutritional scarcity by adopting a starving survival stage, a precursor to the VBNC condition [14,15]. The extensive distribution and strong survival ability of *A. hydrophila* renders it a notable emerging public health threat, particularly considering its potential to enter the VBNC state in response to environmental stressors.

In the context of seafood processing and preservation, chemical preservatives are most commonly used to inhibit microbial growth and extend shelf life [16]. Regulatory agencies like the FDA and the European Food Safety Authority (EFSA) have concluded that preservatives such as sodium chloride (NaCl) and sodium benzoate (SB) are “generally recognized as safe” (GRAS) at specific concentrations for the preservation of seafood products [17]. Elevated salinity imposes osmotic stress that disrupts cellular water balance, membrane integrity, and nutrient transport, while sodium benzoate functions primarily as a bacteriostatic preservative: its mode of action involves disrupting intracellular pH balance and interfering with metabolic enzyme activity. Rather than fully inactivating bacteria, this can encourage a transition into a dormant state. Previous studies have demonstrated its effectiveness in reducing water activity, which in turn inhibits the growth of food spoilage microorganisms, slows spoilage-related reactions, and extends the shelf life of fish and other fishery products [17,18]. There is a notable lack of comprehensive research on the specific role of these preservatives, particularly in inducing the VBNC state in foodborne pathogens such as *A. hydrophila.*

Understanding the VBNC state requires integrated physiological, structural, and molecular investigations, including plate count-based culturability assessment; flow cytometry-based viability profiling, as it enables rapid discrimination between live, injured, and non-culturable cells based on membrane integrity and metabolic activity [19]; microscopy-based assessment like scanning electron microscopy (SEM) and transmission electron microscopy (TEM) to evaluate morphological adaptation; and omics-based analyses, which provide a multidimensional view of stress-induced dormancy. Previous studies on foodborne pathogens such as *Vibrio parahaemolyticus*, *Vibrio vulnificus*, *Escherichia coli*, and *Staphylococcus aureus* have demonstrated that VBNC induction is accompanied by reductions in cell size, membrane restructuring, altered permeability, differentially expressed genes for metabolism and shifts in metabolic and regulatory pathways that support long-term persistence under environmental stress [19,20,21]. These physiological investigations have also identified key molecular signatures, including changes in amino acid metabolism, nucleotide turnover, osmolyte accumulation, and membrane lipid composition, which are believed to contribute to the maintenance of cellular integrity during dormancy. Importantly, elucidating these adaptive mechanisms is essential for identifying molecular targets and candidate inhibitory compounds capable of preventing VBNC formation. By linking metabolite shifts to physiological resilience, metabolomics-guided characterization can identify key metabolic targets and candidate inhibitory compounds of VBNC formation, thereby improving detection and control of persistent foodborne pathogens [22].

Metabolomics is an analytical approach that enables comprehensive identification and quantification of low-molecular-weight metabolites within a biological sample, thereby providing a direct snapshot of cellular physiological status [23]. Typically, metabolomic profiling is performed using advanced platforms such as nuclear magnetic resonance (NMR) spectroscopy and chromatography-coupled mass spectrometry techniques like LC-MS and GC-MS, which allow for sensitive detection of a broad range of intracellular metabolites and metabolic intermediates. Metabolomic analysis in stress-induced bacteria enables the detection of subtle biochemical alterations through the identification of differentially expressed metabolites, disrupted metabolic pathways, and potential biomarkers, thereby extending beyond conventional assessments of viability [23]. Recent studies on the metabolomic profiling of *Lacticaseibacillus paracasei* revealed significant changes in amino acids, sugars, nucleotides, and lipid metabolites during VBNC induction and recovery, highlighting metabolic remodeling associated with survival under stress. Similarly, investigations of chlorine-induced VBNC *Escherichia coli* demonstrated global metabolic alterations indicative of reduced energy metabolism and adaptive stress responses.

The objective of this present study is to provide a comprehensive physiological and metabolomic characterization of VBNC state induction in *A. hydrophila* under combined osmotic and preservative stress at 4 °C. The rationale for selecting this condition lies in its close relevance to seafood preservation environments, where elevated salinity, chemical preservatives, and low temperature coexist and are known to suppress bacterial culturability without necessarily eliminating viability [24,25], thereby offering mechanistic evidence of survival strategies in VBNC persistence, under realistic seafood preservation environments.

## 2. Materials and Methods

### 2.1. Bacterial Culture Preparation and VBNC Induction

The *A. hydrophila* strain ATCC 7966 was acquired from the American Type Culture Collection (ATCC; Manassas, VA, USA). The bacterial strain was revived from −80 °C glycerol stock cultures prior to experimentation. For preparation of the working culture, a single colony was inoculated into 50 mL of tryptone soya broth (TSB; Sigma-Aldrich, St. Louis, MO, USA) and incubated overnight at 37 °C under aerobic conditions. The working culture was prepared by inoculating the bacteria in 50 mL of tryptone soya broth (TSB; Sigma-Aldrich, St. Louis, MO, USA), followed by overnight incubation at 37 °C. To simulate preservative-associated stress conditions relevant to seafood processing environments, we designed an experimental model combining osmotic stress with a commonly used food preservative, sodium benzoate (SB) (99% purity, KemAus™, New South Wales, Australia). The saline solutions were prepared from sodium chloride (NaCl) (99.9% AR grade, KemAus™, New South Wales, Australia) in deionized water, with concentrations of 0.85% (*w*/*v*) (control) and 4% (*w*/*v*) (treatment), and sterilized by autoclaving at 121 °C for 15 min before use in the induction of the VBNC state. The prepared saline solutions were further supplemented with a fixed concentration of SB (1 g/L) in both conditions to mimic preservative exposure in food preservation. The SB was sterilized by filtration through a 0.22 µm membrane (Sartorius, Göttingen, Germany) before being added to the respective solutions. The overnight-cultured bacterial suspension was harvested by centrifugation (5000× *g* for 10 min), washed twice, and resuspended in sterile physiological saline (0.85% NaCl). The concentration was initially standardized to approximately 10^8^ CFU/mL by adjusting the optical density (OD 600 = 0.08) using a spectrophotometer. This suspension was then serially diluted to a target concentration of 10^6^ CFU/mL in physiological saline before being added to the control and treatment solutions. The inoculated samples were covered with aluminum foil to prevent light penetration and incubated at 4 °C. The survival of the bacteria and the VBNC induction state were monitored using the plate count method, flow cytometry and fluorescent microscopy analyses. All experiments were conducted in triplicate (*n* = 3).

### 2.2. Determination of Culturable Bacterial Count

The culturable bacterial count of *A. hydrophila* was determined at various time intervals to assess bacterial growth and count in induction solutions. Samples were collected at 6-day intervals beginning immediately after inoculation (day 0) and continuing until the establishment of the VBNC state. The plate count method was used to quantify the bacterial population. Briefly, 100 µL of the inoculated solution was taken from decimal dilutions of normal saline and spread in triplicate onto tryptic soy agar (TSA) plates. These plates were then incubated at 37 °C for 24, 48 and 72 h. Colony counts were performed, and viable counts were expressed as CFU/mL. Samples of 0.1 mL of undiluted bacterial suspension were plated in triplicate. The transition to the VBNC state was defined when the culturable count dropped below the Limit of Detection (LOD) of 0.1 CFU/mL, calculated based on the 0.1 mL plating volume with no observable colonies after 48 h of incubation at 37 °C, while the viable cells remained detectable by flow cytometry and fluorescence microscopy analyses.

### 2.3. Determination of Cell Viability

Total cell count and viability of *A. hydrophila* were assessed using a FACSCalibur flow cytometer (Becton Dickinson, Milan, Italy), with an Argon laser at 488 nm. Cells were stained using the LIVE/DEAD™ BacLight™ Bacterial Viability Kit (L7012; Invitrogen, Eugene, OR, USA) according to the manufacturer’s protocol. Cell viability was determined using the kit via a direct viability count with two nucleic acid stains, SYTO 9 and propidium iodide (PI). SYTO 9 permeates both live and dead cells, emitting green fluorescence upon binding to DNA, while PI can only enter cells with damaged membranes, replacing SYTO 9 and causing red fluorescence. The fluorescence intensity of the stained bacteria was measured, with green fluorescence collected on the FL1 channel (525 nm) and red fluorescence on the FL3 channel (>670 nm), and the data were subjected to multiparametric analysis. Data were collected as logarithmic signals and analyzed using BD CellQuest^TM^ Pro Version 6.0 software. A total of 100,000 events were acquired for each sample during data collection. The ratio of dead to live cells was also confirmed through this flow cytometry. Based on unstained and single-stained reference samples, selective passages were performed to exclude background noise and debris. The fluorescent image data collection for live cells (green fluorescence) and dead cells (red fluorescence) was observed using fluorescence microscopy (Zeiss Axio Observer, Carl Zeiss Microscopy Deutschland GmbH, Jena, Germany).

### 2.4. SEM-Based Morphological Observation of A. hydrophila

The surface morphology of *A. hydrophila* in normal, VBNC, and dead cells was analyzed using an FEI Quanta 400 scanning electron microscope (FEI, Brno, Czech Republic). The effectiveness of the imposed stressors was verified using defined controls: viable *A. hydrophila* cells maintained in 0.85% NaCl served as the positive control, whereas cells exposed to 100% ethanol for 30 min at room temperature were used as the negative control to represent complete cellular inactivation. The cells including control and treatment conditions were collected by centrifugation at 2320× *g* for 10 min at 4 °C, washed with 0.1 M phosphate-buffered saline (PBS) (pH 7.2), and allowed to adhere to poly-L-lysine-coated glass slides. The adhered cells were fixed with 2.5% glutaraldehyde for 2 h at room temperature, followed by a series of thorough washes with sterile PBS and sterile distilled water. The fixed cells were then dehydrated using a graded ethanol series (50–100%) and subjected to critical-point drying. Finally, the samples were sputter-coated with gold for 1 min and then visualized. The morphological changes in *A. hydrophila* were examined at an acceleration voltage of 20.00 kV and a magnification of 15,000×. The working distance (WD) was maintained at 7.0 mm, with a horizontal field width (HFW) of 8.53 µm.

### 2.5. Determination of Metabolites in A. hydrophila

#### 2.5.1. Sample Preparation for Metabolomics Analysis

*A. hydrophila* cultures subjected to sodium benzoate-induced stress were sampled at 6-day intervals (days 0, 6, 12, 18, 24, and 30). At each time point, bacterial cells were harvested by centrifugation, and the resulting cell pellets were immediately processed for metabolomics extraction. Each cell pellet was transferred into a sterile microcentrifuge tube and resuspended in 200 μL of deionized water (LC-MS–grade, CHROMASOLV, Honeywell, Wunstorfer Strasse, Seelze, Germany). Cell disruption was performed using a probe sonicator (Vibra-Cell VCX750, Fisher Scientific, Pittsburgh, PA, USA) at 60% amplitude with 0.5 s on/off cycles for a total duration of 5 min, while samples were maintained on ice to prevent metabolite degradation. The lysates were centrifuged at 14,000× *g* for 30 min at 4 °C, and the resulting supernatants were carefully collected. For protein precipitation, 300 μL of ice-cold methanol (Wunstorfer Strasse, Seelze, Germany) was added to each supernatant, followed by vortex mixing and incubation at −20 °C for 2 h. Samples were subsequently centrifuged at 14,000 rpm for 15 min, and the clarified supernatants were dried under a gentle stream of nitrogen using a nitrogen evaporator (Organomation, Berlin, MA, USA). The dried extracts were reconstituted in 250 μL of 0.1% formic acid in LC–MS-grade deionized water. Prior to analysis, samples were filtered through 0.45 μm hydrophilic nylon syringe filters, and 100 μL of the filtrate was transferred into LC glass vials with inserts for UHPLC—MS/MS analysis.

#### 2.5.2. UHPLC-ESI-QTOF-MS Analysis

Metabolomic profiling was performed using ultra-high-performance liquid chromatography coupled with electrospray ionization quadrupole time-of-flight mass spectrometry (UHPLC-ESI-QTOF-MS). Chromatographic separation was achieved on a Dionex Ultimate 3000 UHPLC system (Thermo Fisher Scientific, Germering, Germany) equipped with an Acclaim Advantage II C18 column (2.1 × 100 mm, 3 μm) and a corresponding C18 guard column (3 × 10 mm, 5 μm). The injection volume was 3 μL, with the column and autosampler temperatures maintained at 40 °C and 10 °C, respectively. Mass detection was performed using a Bruker compact QTOF mass spectrometer (Bruker Daltonics, Bremen, Germany), operating in both positive and negative ionization modes over an *m*/*z* range of 50–1000. The nebulizer gas pressure, drying gas flow, and drying gas temperature were set to 2 bars, 8 L/min, and 220 °C, respectively. Mobile phase A consisted of water with 0.1% formic acid, while mobile phase B comprised acetonitrile with 0.1% formic acid. Chromatographic separation was conducted using a gradient elution at a flow rate of 0.25–0.35 mL/min, as follows: 0–2 min, 99% A; 2–17 min, linear gradient to 99% B; 17–20 min, 99% B; 20–20.1 min, return to 99% A; 20.1–30 min, re-equilibration at 99% A. Sodium formate was used for external mass calibration. Data acquisition included an auto-MS scan (0–0.3 min) for calibration and an auto-MS/MS scan (0.3–30 min) for metabolite fragmentation, with a scan frequency of 12 Hz. The precursor ion isolation width was set to 0.5 Da, with a maximum of three precursors per cycle, a cycle time of 0.5 s, and an intensity threshold of 400 counts. Active exclusion was applied after three spectra and released after 0.2 min.

#### 2.5.3. Data Processing and Metabolite Identification

Raw LC–MS data were processed using MetaboScape^®^ 2022 (Bruker Daltonics, Bremen, Germany) according to previously established workflows and parameter settings [26,27]. Feature extraction was conducted using the T-ReX 3D algorithm with an intensity threshold of 1000, a minimum peak length of seven spectra, and peak area integration for quantitative analysis. Internal mass calibration was applied within the retention time window of 0.1–0.3 min to ensure mass accuracy. MS/MS spectra were acquired using the average acquisition method over a retention time range of 0.3–25 min and a mass range of *m*/*z* 50–1000. Metabolite annotation was achieved through MS/MS spectral matching and retention time alignment against Bruker MetaboBASE Personal Library 2.0, an extensively curated metabolomics reference database. Compounds with high-confidence MS/MS matches were retained as identified metabolites for subsequent analysis.

### 2.6. Statistical and Bioinformatic Analysis of Metabolomic Data

The processed metabolomics data were exported as comma-separated value (CSV) files and imported into MetaboAnalyst 6.0 (McGill University, Montreal, QC, Canada), a web-based platform for metabolomics data processing and statistical analysis [26]. All detected intracellular metabolites were initially uploaded and subjected to data integrity checks, including missing value assessment, data normalization, and compound name matching against the MetaboAnalyst reference database. To ensure statistical stability for metabolites below the detection limit, zero values were handled via data imputation by replacing them with half of the minimum positive intensity detected in the dataset [28]. This was followed by log-transformation and auto-scaling to normalize the distribution, allowing for the calculation of stable fold-change values and preventing undefined results [29].

Subsequently, the curated datasets were analyzed using the Statistical Analysis (one factor) module to evaluate time-dependent metabolic variations under sodium benzoate-induced stress conditions. Multivariate analyses, including principal component analysis (PCA), were performed to examine temporal clustering patterns and metabolic trajectory shifts associated with VBNC induction. Hierarchical clustering analysis (HCA) was conducted using Euclidean distance and Ward’s linkage method to visualize similarities among sampling time points and identify coordinated metabolite expression patterns [30]. In parallel, volcano plot analysis was performed to identify significantly altered metabolites between selected time points and end points based on log2 fold change and adjusted *p*-values. Univariate statistical testing was applied using Student’s *t*-test/one-way ANOVA, as appropriate, with false discovery rate (FDR) correction, and metabolites with *p* < 0.05 were considered statistically significant. Significantly altered metabolites of the control and treatments were further subjected to pathway enrichment analysis using the Pathway analysis module and Enrichment Analysis module in MetaboAnalyst to identify key metabolic pathways associated with stress adaptation and VBNC state formation in *A. hydrophila*.

### 2.7. Statistical Analysis

Statistical analysis was performed for culturability and viability assessment techniques using two-way analysis of variance (ANOVA), with mean comparisons evaluated through Tukey’s test. A *p*-value of less than 0.05 was considered statistically significant.

## 3. Results

### 3.1. Time-Dependent Loss of Culturability During VBNC Formation

**Figure 1:**
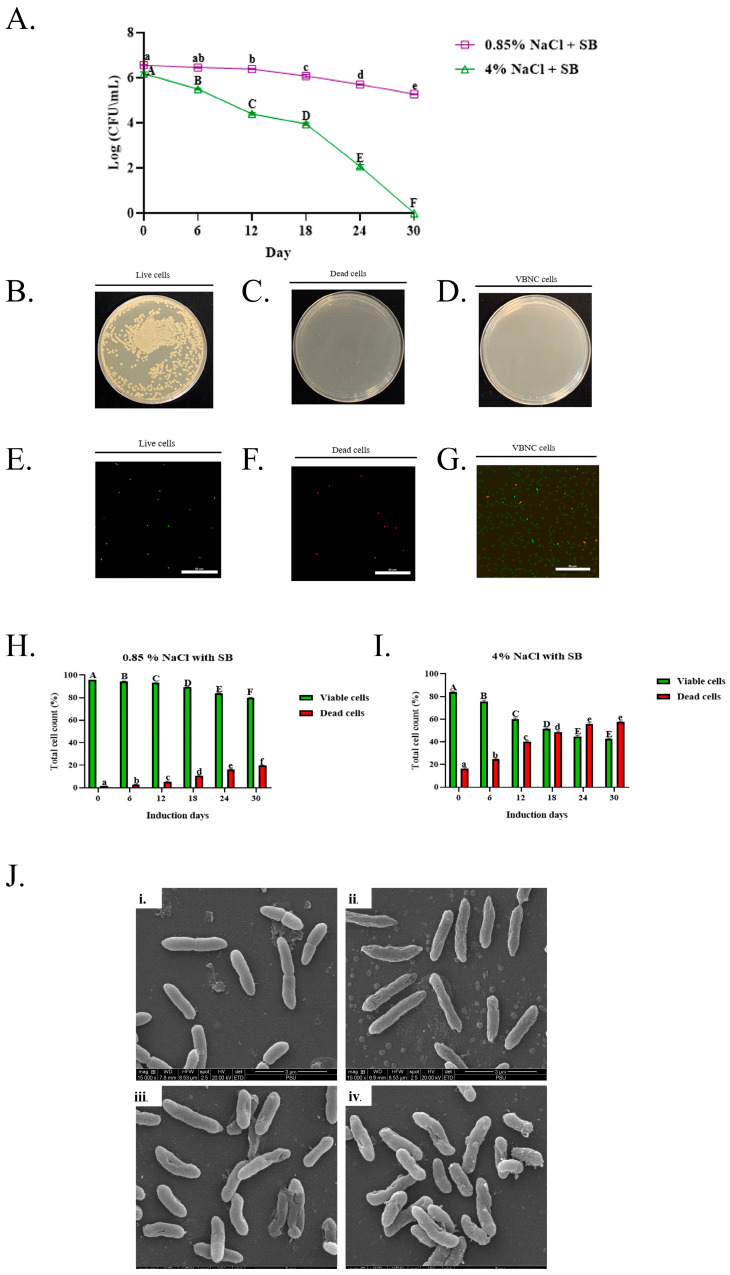
TPC analysis, SEM imaging, colony morphology, fluorescent staining, and flow cytometric analysis of *A. hydrophila*. (**A**): Results of time-course changes in culturability of *A. hydrophila*. (**B**,**E**): Colony morphology and fluorescence microscopy analysis of live cells. (**C**,**F**): Colony morphology and fluorescence microscopy analysis of dead cells. (**D**,**G**): Colony morphology and fluorescence microscopy analysis of VBNC cells. (**H**,**I**): Viability assessment of *A. hydrophila* in 0.85% NaCl with SB and 4% NaCl with SB. (**J**): Results of morphological and structural changes by SEM, (**i**) normal live cell control, (**ii**) normal dead cell control, (**iii**) 0.85% NaCl with SB at day 30, (**iv**) 4% NaCl with SB at day 30. Values are expressed as mean ± SD (*n* = 3) for TPC and viability assessment; different letters within the same group indicate significant differences between induction days (*p* value < 0.05) based on Tukey’s multiple comparisons test. Upper case letters in TPC and viability assessment represent treatment group and viable cells, while lower case letters are for control group and dead cells.

The time-course changes in culturability of *A. hydrophila* under stress are illustrated by total plate count (TPC) analysis (Figure 1A and Supplementary Table S1). Under 0.85% NaCl with SB, bacterial cells remained culturable throughout the 30-day incubation at 4 °C, exhibiting only a gradual decline in viable counts. The mean population decreased steadily from approximately 6.6 log CFU/mL on day 0 to about 5.3 log CFU/mL by day 30. In contrast, exposure to 4% NaCl with SB resulted in a rapid and pronounced reduction in viable cell counts over time. Following an initial decrease from 6.2 log CFU/mL at day 0 to 4.4 log CFU/mL by day 12, culturability declined sharply after day 18. By day 24, viable counts dropped to approximately 2.0 log CFU/mL, and complete loss of detectable colonies was observed at day 30, below the Limit of Detection (LOD) of 10 CFU/mL (1 log CFU/mL). In the control group, culturable *A. hydrophila* cells produced well-defined colonies on TSA plates (Figure 1B). No additional colonies were observed after the initial 24 h incubation period on day 30. Correspondingly, fluorescence microscopy revealed numerous viable cells emitting green fluorescence (Figure 1E). In contrast, dead cells showed no colony formation on agar plates (Figure 1C) and exhibited predominantly red fluorescence signals (Figure 1F). Notably, cells induced into the VBNC state did not form visible colonies (Figure 1D), but fluorescence imaging demonstrated a substantial population of green fluorescent cells (Figure 1G), indicating that these cells remained viable but non-culturable.

Flow cytometry is a powerful technique for assessing bacterial viability at the single-cell level [31]. In this study, flow cytometric analysis was applied alongside the TPC method to monitor the induction and progression of the VBNC state in *A. hydrophila* under preservative-associated stress conditions. Under 0.85% NaCl with SB, at day 0, the mean live-cell percentage was 95.93 ± 0.01%, which slightly decreased to 94.47 ± 0.25% at day 6 and 93.00 ± 0.30% on day 12. A moderate reduction was observed at later stages, with live-cell percentages of 89.17 ± 0.55% on day 18 and 83.77 ± 0.55% on day 24. By day 30, viability remained at 80.07 ± 0.25%. For 4% NaCl with SB, at day 0, the live-cell percentage was 83.67 ± 0.68%, which declined substantially to 75.57 ± 0.80% on day 6. By day 12, it was 60.03 ± 0.95% and continued to decrease to 51.27 ± 0.90% on day 18. On day 24, viability reached 44.43 ± 1.05% and finally to a live-cell percentage of 42.50 ± 0.80% on day 30; however, total plate count (TPC) showed no colony formation, confirming the transition into the VBNC state (Figure 1I and Supplementary Table S1). The total cell counts (cells/mL), under both control and treatment conditions, are provided in Supplementary Table S1.

### 3.2. Morphological Analysis of A. hydrophila

Upon entering the VBNC state, *A. hydrophila* undergoes significant morphological and structural changes, as observed by SEM (Figure 1B). Normal live cells of *A. hydrophila* control samples were found to be typically rod-shaped with rounded ends and randomly distributed (Figure 1(Ji)). Control samples of dead cells exhibited severe surface damage, including rupture, shrinkage and aggregation (Figure 1(Jii)). The morphological changes observed were directly correlated with the concentration of inducing agents. The control group of untreated cells maintained their characteristic smooth, short rod shape, but the cells treated with 0.85% NaCl with SB showed some surface wrinkles on them (Figure 1(Jiii)). Cells exposed to a higher concentration of 4% NaCl with SB showed structural modifications, including becoming a short rod shape, developing a rough surface, and becoming more shriveled, sunken, wrinkled and concave on their surfaces (Figure 1(Jiv)).

### 3.3. Time-Dependent Metabolic Trajectories

#### 3.3.1. Time-Resolved PCA of *A. hydrophila*

**Figure 2:**
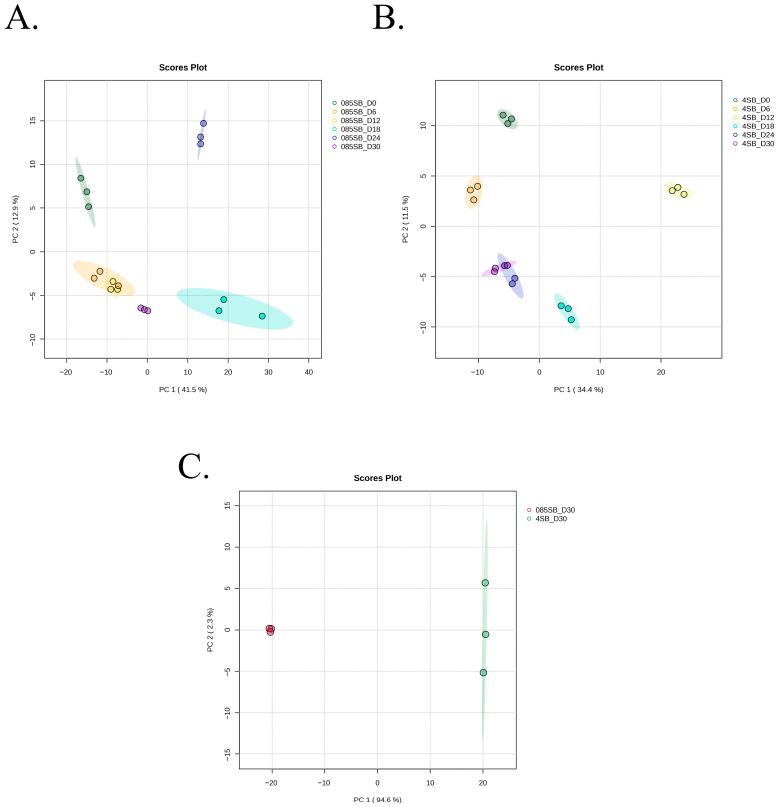
PCA score plot showing time-dependent metabolic trajectories. (**A**): PCA score plot under 0.85% NaCl with SB contributing to time-dependent metabolic changes under 0.85% NaCl with SB. (**B**): PCA score plot under 4% NaCl with SB contributing to time-dependent metabolic changes under 4% NaCl with SB. (**C**): PCA score plot of control and treatment at VBNC state (day 30). Note: 085_SB—0.85% NaCl with SB (Control group) and 4_SB—4% NaCl with SB (Treatment group).

Principal component analysis (PCA) of metabolomic profiles collected over 30 days under 0.85% NaCl with SB (control) demonstrated a structured but moderate temporal shift in the global metabolome while cells remained culturable. The first two principal components explained 54.4% of the total variance (PC1: 41.5%; PC2: 12.9%) (Figure 2A) with observed group separation that was further supported by PERMANOVA analysis in Supplementary Figure S1. Day 0 samples formed a clearly separated cluster along the negative axis of PC1. Subsequent time points (day 6–day 18) exhibited gradual displacement toward intermediate positions. Day 24 samples clustered distinctly along the positive direction of PC2, while day 30 samples grouped closer to intermediate time points but remained separated from day 0. In contrast, PCA of 4% NaCl with SB (treatment) showed a more pronounced and directional metabolic divergence associated with VBNC induction. The first two components accounted for 45.9% of the variance (PC1: 34.4%; PC2: 11.5%) (Figure 2B), and the observed group separation was also supported by PERMANOVA analysis in Supplementary Figure S2. Day 0 samples clustered distinctly from all subsequent time points. A progressive and ordered displacement of samples was observed along PC1 from day 6 through day 18. Notably, day 24 and day 30 formed tight, well-defined clusters separated from earlier stages, with day 30 occupying an extreme position in the PCA plot, whereas PCA comparing day 30 samples of the culturable control and the VBNC-inducing treatment demonstrated a clear and complete separation between groups along PC1, which accounted for 94.6% of the total variance (Figure 2C). The control samples clustered tightly on the negative side of PC1, whereas the VBNC treatment samples formed a distinct cluster on the positive axis, indicating PCA shows strong metabolic divergence coincidence with the different conditions. Together, these PCA results demonstrate that both culturable and VBNC conditions in *A. hydrophila* exhibit time-dependent metabolic trajectories and directional shifts based on the induced conditions.

#### 3.3.2. Heat Map Analysis of Time-Dependent Metabolic Remodeling

**Figure 3:**
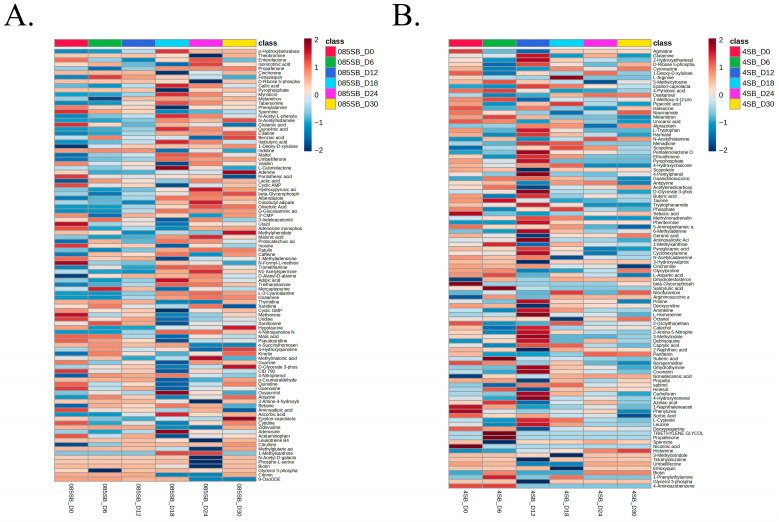
Heat map analysis. (**A**): Heat map analysis highlighting metabolites contributing to time-dependent metabolic changes under 0.85% NaCl with SB (Control). (**B**) Heat map analysis highlighting metabolites contributing to time-dependent metabolic changes under 4% NaCl with SB (treatment). Heat map showing hierarchical clustering of significantly altered metabolites (*t*-test/ANOVA, *p* < 0.05) across different time points (day 0, 6, 12, 18, 24, and 30) during storage at 4 °C. Metabolite intensities were z-score-normalized, and clustering was performed using Euclidean distance with Ward’s linkage method. Colors represent relative metabolite abundance (red, higher; blue, lower). Note: 085_SB—control group and 4_SB—treatment group.

Heat map analysis of the top significantly altered metabolites across the storage period (day 0–day 30) for the control group demonstrated a structured, time-dependent metabolic trajectory in *A. hydrophila*. The metabolites included were selected based on one-way ANOVA followed by pairwise *t*-tests (*p* < 0.05), highlighting statistically robust temporal variation (Figure 3A) and statistically correlating with the PCA plot of the control group (Supplementary Table S2 and Supplementary Figure S3). Early time points (day 0 and day 6) clustered together and were characterized by relatively higher abundance of key amino acids and organic acids, including Phenylalanine, Spermine, and Glutamic acid, which gradually decreased at later time periods. A significant metabolic shift occurred during the intermediate phase (day 12–day 18), marked by the downregulation of several nucleotide-related compounds such as Uracil and Adenosine monophosphate, alongside a sharp decrease in Malonic acid. By the late storage stage (day 24–day 30), the metabolic profile shifted toward the accumulation of stress-associated metabolites and degradation products, including hypotaurine, Methylmalonic acid, and Epsilon-caprolactam.

Heat map visualization of significantly altered metabolites for the treatment group (Figure 3B), together with the consistent clustering pattern observed in the corresponding PCA plots (Supplementary Table S2 and Supplementary Figure S4), demonstrated that the early-to-mid time points (day 0–day 12) were marked by a sharp upregulation of several amino acids and their derivatives, such as L-Arginine, L-Tryptophan, and L-Homoserine, alongside a prominent increase in Xanthine and Cytidine levels, suggesting an initial compensatory response to the treatment stress. In the late induction phase (day 18–day 24), a substantial shift was observed, indicated by the elevated intensities of Sebacic acid, Caprylic acid, and glycerol-3-phosphate. The metabolic profile at day 30 was uniquely defined by the high abundance of Biotin, Epsilon-caprolactam, and N-Acetylhistamine, while primary metabolic intermediates such as D-Ribose 5-phosphate and Pyroglutamic acid were significantly downregulated. Collectively, the heat map analysis revealed a clear contrast between the metabolomic profiles of *A. hydrophila* maintained under culturable control and VBNC-inducing treatment groups.

### 3.4. End Point-Based Metabolite Changes

**Figure 4:**
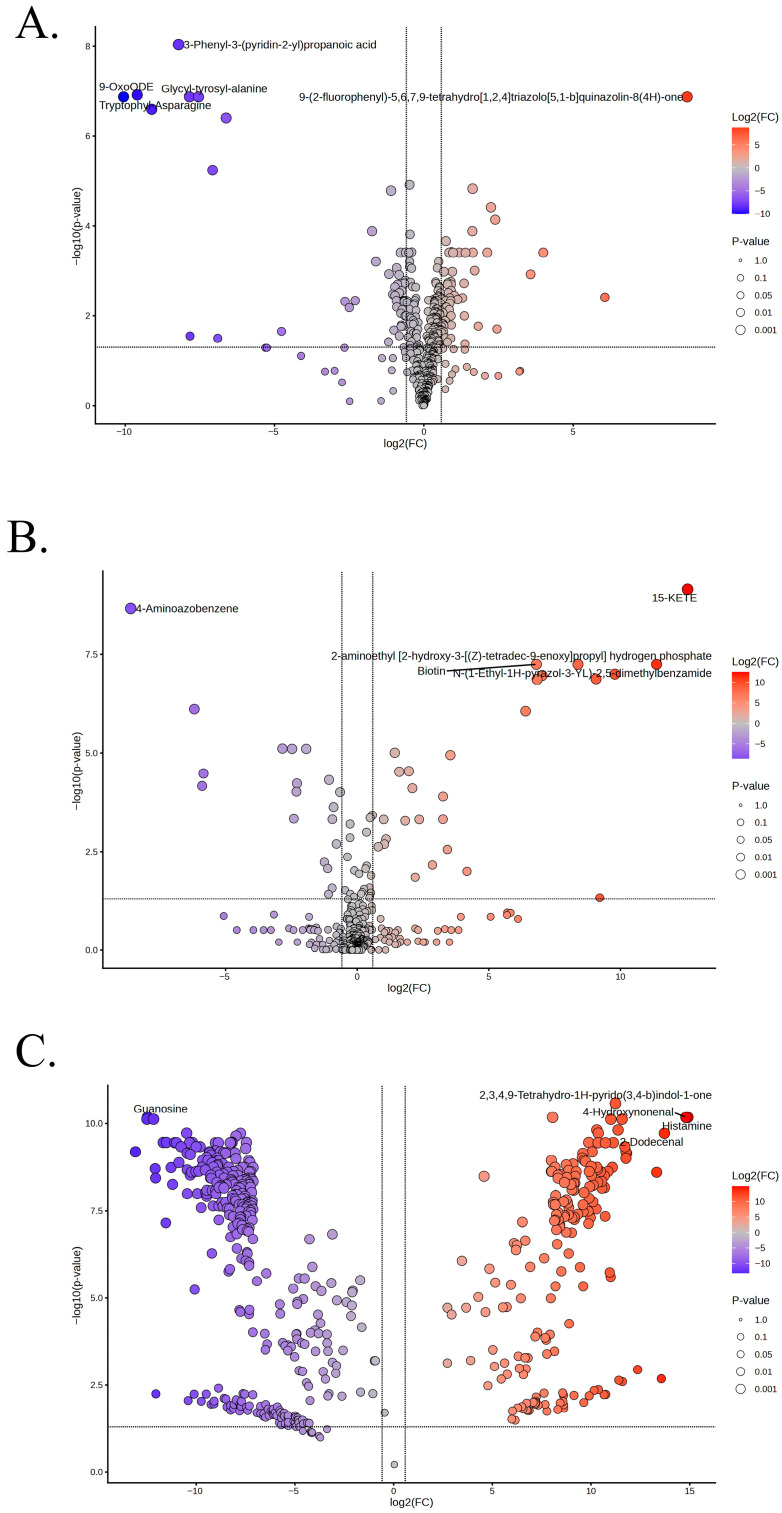
Volcano plot analysis showing differential metabolites in *A. hydrophila*. (**A**): End point analysis of control (day 30/day 0). (**B**): End point analysis of treatment (day 30/day 0). (**C**): End point analysis of treatment and control (day 30/day 30). The *x*-axis represents log2 fold change (D30/D0), and the *y*-axis represents −log10(*p*-value). Significantly altered metabolites exceeding the defined fold-change and significance thresholds are highlighted and labeled.

The comparison of day 30 to day 0 (D30/D0) under control conditions revealed distinct metabolite-level alterations in the volcano plot analysis despite the overall metabolic stability observed in this culturable condition (Figure 4A). Among the significantly upregulated metabolites at day 30, several compounds exhibited large positive log2 fold-change values with strong statistical support. Out of all detected features, 55 metabolites were significantly upregulated, and 46 metabolites were significantly downregulated, while the majority (368 metabolites) showed no statistically significant change (Supplementary Table S3). Among the five most significant metabolites, 9-oxoODE, glycyl-tyrosyl-alanine, tryptophylasparagine and 3-phenyl-3-(pyridin-2-yl) propanoic acid were downregulated and 9-(2-fluorophenyl)-5,6,7,9-tetrahydro[1,2,4]triazolo[5,1-b]quinazolin-8(4H)-one was upregulated at day 30 relative to day 0. Volcano plot analysis for the treatment group, in which the population reached a VBNC state by day 30, showed distinct metabolomic changes when day 30 (D30) was compared with day 0 (D0) (Figure 4B) (Supplementary Table S3). A total of 28 metabolites were significantly upregulated, and 15 metabolites were significantly downregulated, while 428 metabolites remained statistically insignificant. The top five prominent annotated up- and downregulated metabolites included four upregulated compounds, namely 15-KETE, biotin, 2-aminoethyl [2-hydroxy-3-(Z)-tetradec-9-enoyl] oxy], and propyl hydrogen phosphate, and one downregulated compound, 4-Aminoazobenzene, all showing large positive fold changes and high statistical significance. These metabolites represent the most pronounced metabolic increases associated with the VBNC transition under 4% saline with SB.

Analysis of samples on the day of VBNC induction (day 30) from the treatment and culturable control revealed extensive metabolic reprogramming (Figure 4C) (Supplementary Table S3). Out of the detected features, 518 metabolites were significantly altered, comprising 336 significantly downregulated metabolites and 182 significantly upregulated metabolites, while only 14 metabolites remained statistically non-significant. Notably, guanosine was among the most strongly downregulated metabolites on day 30 under VBNC-inducing conditions. In contrast, a distinct subset of metabolites displayed high positive log2 fold-change values with strong statistical significance. Prominently upregulated metabolites included 4-hydroxynonenal, histamine, 2-dodecenal, and 2,3,4,9-tetrahydro-1H-pyrido(3,4-b) indol-1-one, which clustered in the upper right quadrant of the plot.

### 3.5. Pathway-Level Metabolic Reprogramming During VBNC Induction

**Figure 5:**
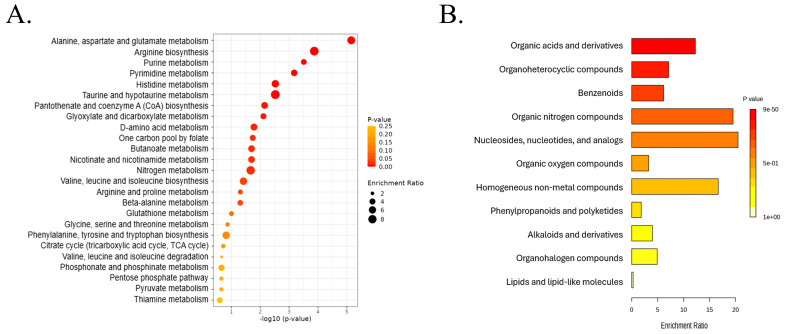
Pathway analysis and metabolite category enrichment analysis. (**A**): Top pathways of significantly altered metabolites identified in *A. hydrophila* during VBNC induction. The *x*-axis represents −log10(*p*-value), indicating pathway significance, while dot size corresponds to the enrichment ratio and color intensity reflects adjusted *p*-value. (**B**): Top metabolite categories of significantly altered metabolites identified in *A. hydrophila* during VBNC induction. The *x*-axis represents −log10(*p*-value), indicating significance, and color intensity reflects the adjusted *p*-value.

Pathway over-representation analysis of the significantly altered metabolites identified in the comparison between the treatment and control group on the day of VBNC induction was carried out (Figure 5A and Supplementary Table S4). The *E. coli* KEGG pathway database was selected as the reference because *A. hydrophila* currently lacks a fully curated and comprehensive pathway annotation in KEGG comparable to model organisms. As both belong to the class Gammaproteobacteria and they share highly conserved core metabolic networks, including central carbon metabolism, amino acid biosynthesis, nucleotide metabolism, and stress-response pathways, and makes it a reliable framework for pathway mapping.

The most significantly enriched pathway was alanine, aspartate and glutamate metabolism, with 8 hits out of 28 pathway metabolites, showing strong statistical significance (enrichment *p* = 7.03 × 10^−6^; FDR = 5.7 × 10^−4^). Arginine biosynthesis was also highly enriched (5/14 hits, enrichment *p* = 1.34 × 10^−4^; FDR = 5.44 × 10^−3^), whereas nucleotide metabolism was prominently affected, as reflected by significant enrichment of purine metabolism (10/70 hits, enrichment *p* = 3.09 × 10^−4;^ FDR = 8.34 × 10^−3^) and pyrimidine metabolism (7/39 hits, enrichment *p* = 6.65 × 10^−4^; FDR = 1.35 × 10^−2^). Additionally, histidine metabolism (4/16 hits, enrichment *p* = 2.98 × 10^−3^; FDR = 4.04 × 10^−2^) and taurine and hypotaurine metabolism (3/8 hits, enrichment *p* = 2.99 × 10^−3^; FDR = 4.04 × 10^−2^) were significantly over-represented. Several energy- and cofactor-related pathways, including pantothenate and CoA biosynthesis (4/20 hits, enrichment *p* = 7.04 × 10^−3^) and glyoxylate and dicarboxylate metabolism (5/32 hits, enrichment *p* = 7.72 × 10^−3^), showed moderate enrichment but did not remain significant after FDR correction, suggesting secondary metabolic adjustments. In contrast, the tricarboxylic acid (TCA) cycle, glycolysis/gluconeogenesis, and fatty acid biosynthesis exhibited minimal enrichment and high FDR values, with entry into a VBNC state. This metabolic signature reflects a shift toward survival-oriented biochemical pathways supporting stress tolerance, macromolecular maintenance, and metabolic quiescence during prolonged exposure to high-salinity sodium benzoate conditions.

### 3.6. Metabolite Category Distribution During VBNC Induction

Category-based over-representation analysis was performed on the significantly altered metabolites identified in the comparison between the control and treatment group on the day of VBNC induction, and it revealed strong and selective enrichment of multiple metabolite classes (Figure 5B and Supplementary Table S4). The most prominently enriched category was organic acids and derivatives (64 hits, enrichment *p* = 8.51 × 10^−50^; FDR = 2.98 × 10^−48^), indicating extensive remodeling of central metabolic intermediates during the VBNC transition. This was followed by significant enrichment of organoheterocyclic compounds (53 hits, enrichment *p* = 1.73 × 10^−29^; FDR = 3.03 × 10^−28^) and benzenoids (35 hits, enrichment *p* = 1.54 × 10^−17^; FDR = 1.79 × 10^−16^), reflecting pronounced alterations in aromatic and heterocyclic metabolite pools. Organic nitrogen compounds (14 hits, enrichment *p* = 3.41 × 10^−14^; FDR = 2.98 × 10^−13^) and nucleosides, nucleotides, and analogs (13 hits, enrichment *p* = 1.58 × 10^−13^; FDR = 1.10 × 10^−12^) were also significantly over-represented. Moderate but statistically significant enrichment was observed for organic oxygen compounds (12 hits, enrichment *p* = 3.85 × 10^−4^; FDR = 2.25 × 10^−3^) and homogeneous non-metal compounds (2 hits, enrichment *p* = 6.59 × 10^−3^; FDR = 0.033). In contrast, phenylpropanoids and polyketides; alkaloids and derivatives; organohalogen compounds; and lipids and lipid-like molecules did not show significant enrichment after multiple-testing correction, indicating that lipid metabolism was comparatively conserved during VBNC induction. Collectively, the enrichment profile demonstrates that VBNC induction in *A. hydrophila* is characterized by selective over-representation of metabolites linked to organic acid metabolism, nucleotide dynamics, nitrogen handling, and chemically complex stress-associated compounds, rather than broad changes across all metabolite classes.

### 3.7. Potential Biomarker Analysis

**Figure 6:**
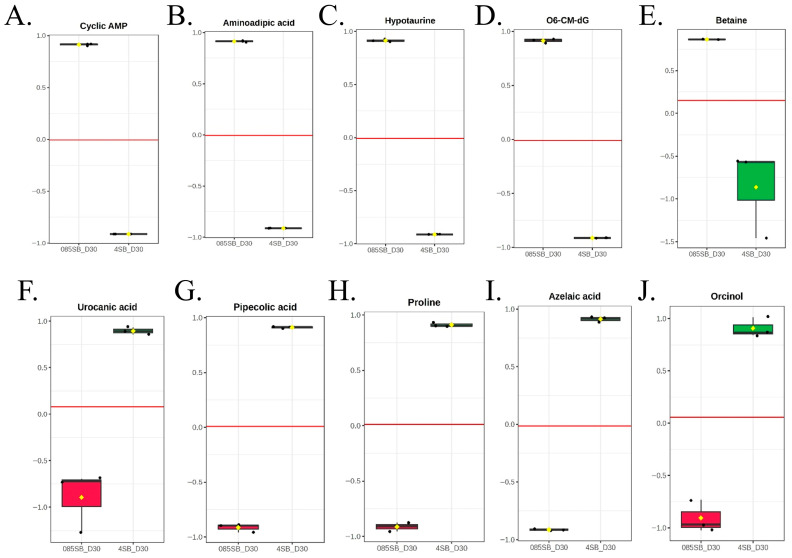
Biomarker analysis distinguishing culturable and VBNC states of *A. hydrophila*. Box plots showing normalized abundances of key discriminatory metabolites at the day 30 culturable state and VBNC state. (**A**) Cyclic AMP, (**B**) aminoadipic acid, (**C**) hypotaurine, (**D**) O6-CM-dG, (**E**) betaine, (**F**) urocanic acid, (**G**) pipecolic acid, (**H**) proline, (**I**) azelaic acid, and (**J**) orcinol. Central lines represent medians, boxes indicate interquartile ranges, and whiskers denote variability within groups. Red reference lines indicate zero-centered normalized expression. The *x*-axis represents the two comparison groups (0.85% NaCl with SB—control and 4% NaCl with SB—treatment, at day 30), while the *y*-axis depicts normalized and auto-scaled metabolite intensities.

To identify metabolic biomarkers associated with the VBNC state of *A. hydrophila*, a combined analytical strategy integrating significant metabolites at VBNC induction, mean intensity and fold change (FC) was employed to compare VBNC cells induced against culturable controls. A list of candidate metabolites were found (Supplementary Table S3) and showed strong statistical significance, and among them, we selected 10 suitable metabolites that are mechanistically linked to osmotic stress adaptation, redox balance, nucleotide suppression, and metabolic reprogramming characteristic of the VBNC state [23,32,33] (Table 1) (Figure 6A–J).

Among these biomarkers, five metabolites were markedly downregulated in VBNC cells, including cyclic AMP, aminoadipic acid, hypotaurine, O6-CM-dG, and betaine (Figure 6A–E). These metabolites exhibited high mean intensities, with fold-change values ranging from 4.5 to 11.19. Conversely, five metabolites were significantly upregulated in VBNC cells, namely urocanic acid, pipecolic acid, proline, azelaic acid, and orcinol, with fold changes ranging from 5.1 to 9.97 (Figure 6F–J). Collectively, this biomarker panel captures key biochemical hallmarks of VBNC physiology in *A. hydrophila*, characterized by suppression of growth-related signaling and osmolyte metabolism alongside activation of stress tolerance and metabolic conservation pathways.

## 4. Discussion

*A. hydrophila*, an important foodborne and aquatic pathogen, can undergo profound metabolic and physiological reprogramming to enter a VBNC state under preservative-associated saline stress, highlighting a critical survival strategy that may enable persistence and retain pathogenic potential in seafood and food-processing environments. The combined exposure to 4% NaCl with SB and 4 °C creates a multifactorial stress environment that mechanistically drives *A. hydrophila* into the VBNC state. High salinity imposes hyperosmotic stress, causing rapid water efflux, cytoplasmic dehydration, increased ionic strength, and membrane tension, which disrupt protein conformation and central metabolism [46]. Cold temperature further reduces membrane fluidity and enzymatic activity, limiting ATP generation and slowing biosynthetic processes, which has also been reported in *Shewanella putrefaciens*, *Poseidonibacter antarcticus* and *Bacillus cereus* [47,48,49,50].

The bacteriostatic efficacy of SB is pH-dependent, as only the undissociated form of benzoic acid (*pKa* = 4.19) can permeate the lipid bilayer of *A. hydrophila*, likely in processed seafood such as marinated fish, pickled shrimp, or seafood salads where acidulants (citric or acetic acid) typically lower the pH; thus, benzoic acid effectively diffuses into the cytoplasm. Once inside, it dissociates and releases protons, forcing the cell to expend significant energy to maintain pH homeostasis and causing the partial collapse of the proton motive force [51,52]. For a psychrotrophic pathogen like *A. hydrophila*, this internal acidification inhibits critical metabolic enzymes and disrupts redox balance [22]. Our findings suggest that the combination of SB, hypersalinity, and cold storage does not necessarily result in microbial inactivation. Instead, it leads to metabolic suppression, driving the pathogen into a persistent VBNC state [53]. This adaptive strategy was also observed for foodborne pathogens like *E. coli* and *V. parahaemolyticus* in preserved seafood products, posing a potential public health risk due to the ability of VBNC cells to evade detection while retaining possible pathogenic potential upon resuscitation [3,54]. In this study, however, the induction of the VBNC state occurred in a laboratory saline environment with a near-neutral pH for the VBNC group [55]. Although the pH was not monitored or adjusted during the 30-day incubation, this approach was intended to evaluate the survivability of foodborne pathogens under the natural, unbuffered conditions of a saline model. While the use of SB is strictly regulated and prohibited in fresh fish within certain jurisdictions, such as the European Union, it remains a vital preservative in the global processing of semi-preserved seafood, including marinated, salted, and vacuum-packaged fish products in Asian countries [17,56]. In this context, the presence of SB in the control and treatment groups allowed for the isolation of salinity as the critical factor in VBNC induction, while SB imposed baseline chemical stress, likely creating the multifactorial stress environment leading to VBNC conditions [57].

The pronounced morphological remodeling observed under 4% NaCl with SB at 4 °C reflects an active and coordinated stress-adaptation response rather than passive cellular deterioration. The high salinity imposes strong hyperosmotic pressure, leading to the rapid efflux of intracellular water, reduced turgor pressure, and cytoplasmic shrinkage as compared to normal live cells [58]. This dehydration effect explains the shortened and coccoid-like morphology, surface wrinkling, and concave structural features visualized by SEM [59]. Such size reduction is a well-recognized adaptive strategy during VBNC induction by stressors, which has been seen in *S. aureus*, *V. vulnificus*, and *Cronobacter sakazakii* as smaller cell forms minimize exposed surface area and reduce metabolic demand, thereby enhancing survival under hostile conditions [60,61,62]. Concurrently, low-temperature exposure (4 °C) would likely decrease membrane fluidity and enzymatic activity, reinforce metabolic slowdown and promote structural stabilization consistent with dormancy development in *A. hydrophila*. The observed aggregation and polymer-like surface layers likely reflect stress-induced extracellular polymeric substance production, which enhance cell–cell adhesion and provide an additional protective barrier against osmotic and preservative stress [3]. However, the assessment of viability was primarily based on membrane integrity via SYTO 9/PI staining. Although this is a standard approach, there is a chance that the “viable” (green-fluorescent) population includes cells that are irreversibly injured or recently dead but still possess transiently intact membranes [63]. Indeed, SYTO9/PI staining has been validated in multiple studies for enumerating viable and non-culturable cells by flow cytometry, providing a practical way for distinguishing viability when culturability is lost [59,62].

The time-resolved metabolomic remodeling observed in *A. hydrophila* during the transition into the VBNC state under 4% NaCl with SB parallels previously described VBNC mechanisms in *E. coli*, *V. vulnificus*, and *L. paracasei*, while reflecting stress-specific adaptations to combined osmotic and preservative pressure [54,59,61]. In our study, heat map and volcano analyses demonstrated significant modulation of amino acid-associated metabolites, including methionine, arginine, L-tryptophan, spermidine, and histamine, during VBNC establishment. Similar elevations in intracellular amino acid pools were reported in VBNC *E. coli*, where perturbations in aminoacyl-tRNA biosynthesis, arginine and proline metabolism, alanine–aspartate–glutamate metabolism, and branched-chain amino acid pathways were linked to proteostasis recovery and dormancy regulation [64,65]. Environmental stressors such as chlorine and oxidative agents would induce protein misfolding and aggregation, compromising proteostasis and promoting entry into dormancy [66,67]. Clearance of protein aggregates during VBNC resuscitation has been directly visualized in *E. coli* by TEM [68], supporting the hypothesis that elevated amino acid pools may reflect intracellular recycling of damaged proteins. Moreover, amino acids function as compatible osmolytes, and their accumulation under high salinity is consistent with osmotic adaptation mechanisms described in Gram-negative bacteria [69,70]. The marked suppression of nucleotide-associated metabolites including guanosine, cytidine, inosine, xanthine derivatives, and phosphate intermediates was particularly visible in direct comparisons between day 30 VBNC cells and culturable controls. Similar reductions in purine and pyrimidine metabolism have been reported in VBNC *V. vulnificus* and *E. coli*, where downregulation of thymidine phosphorylase and purine nucleoside phosphorylase corresponded to decreased DNA replication and transcriptional activity [32,71]. The current study revealed selective upregulation of stress- and membrane-associated metabolites, including 4-hydroxynonenal, glycerol-3-phosphate, and histamine. Lipid peroxidation products such as 4-hydroxynonenal are possible markers of oxidative membrane stress, frequently associated with disinfectants and salt-induced VBNC transitions. Similar membrane remodeling and carbohydrate–lipid metabolic adjustments have been described in cold-induced VBNC *L. paracasei*, where membrane transport and fatty acid modulation were enriched [59]. Downregulation of fatty acid synthesis and β-oxidation pathways, particularly fabG and fadE suppression, during VBNC entry and recovery phases has been reported in transcriptome analysis, indicating coordinated restructuring of membrane lipid homeostasis [61].

The pronounced enrichment of pathways such as alanine, aspartate, and glutamate metabolism, alongside arginine biosynthesis, indicates that *A. hydrophila* reprograms its nitrogen and carbon flux toward cellular maintenance rather than active division. Under the combined pressure of the preservatives, these accumulated amino acids function as chemical chaperones [72]. By increasing the hydration shell around proteins, these compatible solutes prevent the exposure of hydrophobic residues, thereby inhibiting the formation of stress-induced protein aggregates [69,70]. Specifically, the accumulation of glutamate and arginine is critical for the recovery of proteostasis. These metabolites facilitate the disaggregation of misfolded proteins by stabilizing intermediate folding states, allowing endogenous chaperone systems such as DnaK-DnaJ-GrpE to more efficiently refold macromolecular structures damaged by osmotic and acidic stress [64,65]. This metabolic investment ensures that the proteome remains functional during dormancy, a prerequisite for eventual resuscitation. Furthermore, the enrichment of taurine, hypotaurine, and histidine metabolism suggests a dual-layered defense: these metabolites act as antioxidants to scavenge reactive oxygen species (ROS) while simultaneously stabilizing membrane integrity [23,73]. By maintaining the solubility of the proteome and protecting the lipid bilayer, *A. hydrophila* preserves the essential machinery required to survive prolonged preservation hurdles in the VBNC state [74,75].

Concurrently, the relatively weak enrichment of central energy-generating pathways such as glycolysis, the tricarboxylic acid (TCA) cycle, and fatty acid biosynthesis reflects global metabolic downscaling, a vital factor of VBNC physiology in which respiratory flux is suppressed to limit endogenous oxidative stress [23]. The appearance of pantothenate and coenzyme A biosynthesis, and glyoxylate and dicarboxylate metabolism further supports the concept of metabolic downshifting [76]. Rather than a complete shutdown of respiration, the VBNC cells reroute carbon flux through energy-efficient and anaplerotic pathways, enabling survival under nutrient limitation and osmotic stress imposed by high salinity and preservative exposure [77]. Significant enrichment of purine and pyrimidine metabolism defines altered nucleotide turnover in VBNC cells [76], as suppression of nucleotide turnover is associated with reduced DNA replication and transcriptional activity in VBNC *Vibrio* species and *E. coli* cells [32,61,71]. These pathway-level changes indicate that VBNC induction in *A. hydrophila* is governed by an actively regulated metabolic strategy involving selective enhancement of amino acid-mediated stress adaptation and repression of biosynthetic and energy-intensive processes, representing an adaptive, persistence-oriented physiological state rather than passive metabolic deterioration [33].

When *A. hydrophila* entered the VBNC state, category-based enrichment analysis revealed a coordinated and mechanistically meaningful reprogramming of intracellular metabolism consistent with an actively regulated persistence strategy rather than passive metabolic collapse [78]. The strongest enrichment of organic acids and derivatives indicates that these function as key intermediates of the TCA cycle, glyoxylate shunt, and amino acid catabolism, and their accumulation suggests suppression of high-flux oxidative respiration while maintaining carbon-sparing pathways that reduce endogenous ROS generation and sustain minimal maintenance metabolism [79]. Similar rerouting toward glyoxylate- and organic acid-associated metabolism has been demonstrated in chlorine- and starvation-induced VBNC *E. coli* and *Pseudomonas aeruginosa*, where disruption of the glyoxylate cycle compromises VBNC survival and metabolic homeostasis [23,54]. Concurrent enrichment of organoheterocyclic compounds and benzenoids further supports an adaptive stress-response phenotype, as these classes often include redox-active metabolites, aromatic signaling molecules, and xenobiotic-like compounds implicated in oxidative defense, detoxification, and membrane stabilization. Food preservatives like SB are known to disrupt intracellular pH and induce oxidative stress, and accumulation of aromatic and heterocyclic metabolites likely reflects activation of chemical defense and stress-signaling circuits [80,81], consistent with observations in VBNC *E. coli* [33,77]. Significant enrichment of organic nitrogen compounds and nucleosides, nucleotides, and analogs might indicate extensive perturbation of nitrogen balance and nucleotide homeostasis, aligning with suppression of DNA replication [82] and cell division while preserving limited nucleotide turnover for genome maintenance and repair [23]. Downregulation of purine and pyrimidine metabolism is a recognized feature of VBNC cells, reflecting metabolic minimization while maintaining molecular integrity for potential resuscitation [32,71]. Moderate enrichment of organic oxygen compounds and homogeneous non-metal compounds further implicates oxidative stress adaptation, consistent with preservative-induced redox imbalance [83], whereas the lack of enrichment for lipids and lipid-like molecules suggests that VBNC induction in *A. hydrophila* involves selective membrane remodeling rather than global lipid turnover [84], a pattern also reported in salt- and cold-induced VBNC *Lactobacillus* species and *S. aureus* [59,62].

The undissociated form of SB as a stressor in the resulting acidification forces the cell to divert limited ATP reserves toward proton efflux pumps to maintain pH homeostasis, but in a real-world food matrix, the acidity of the product would dictate the rate and depth of this ATP depletion [51,52]. This energy drain triggers a strong depletion of cAMP and is implicated as a regulator of entry into non-culturable states in *E. coli* under nutrient stress and related dormancy phenotypes [34]. In parallel, altered amino acid/nitrogen metabolism like aminoadipic acid and the accumulation of proline and pipecolic acid aligns with a widely reported VBNC feature of the conservation of amino acid pools to support proteostasis recovery via recycling/disaggregation of stress-damaged proteins and osmotic and oxidative protection [35,36,37,38]. VBNC studies in *E. coli* and *V. vulnificus* showed amino acid pathway disruption and improved resuscitation after amino acid supplementation, supporting the idea that intracellular amino acid availability can recover the VBNC state [61,85]. Similarly, the reduction in hypotaurine suggests attenuation of amino acid catabolism and redox buffering capacity, consistent with minimized biosynthetic activity during an unculturable state [39]. Proline is a classic bacterial osmoprotectant/ROS buffer, and its elevation is consistent with adaptation to combined cold and high salinity [43]; conversely, shifts in betaine indicate re-prioritization of osmolyte handling as cells move from active osmoregulation toward a low-flux persistence physiology [41]. The decrease in O6-CM-dG, a guanine DNA adduct, is consistent with VBNC cells reducing replication-associated nucleotide turnover and genotoxic burden while maintaining genome integrity. VBNC is often associated with curtailed DNA/RNA synthesis and increased emphasis on maintenance/repair rather than growth [40]. Finally, increased azelaic acid, a dicarboxylic acid that can arise from lipid oxidative cleavage, and orcinol, a phenolic compound with antimicrobial and redox activity, are consistent with the broader enrichment observed in organic acids and aromatic classes, signatures typically linked to membrane remodeling, oxidative stress chemistry, and chemical defense in persistence states rather than biomass production [44,45].

The untargeted nature of our LC-MS/MS analysis led to the putative identification of a broad range of chemical features. While some annotations matched exogenous compounds, these are interpreted as environmental background or database-matching artifacts common in high-resolution untargeted workflows. However, the metabolites were identified through untargeted MS/MS database matching without verification against an authentic chemical standard; their role must be interpreted with caution and limitations, suggesting that future targeted investigations are needed to confirm the extent of genomic damage in dormant *A. hydrophila* [86]. The utilization of the KEGG database of *E. coli K-12 MG1655* as a primary reference for pathway enrichment is a well-established strategy in microbial metabolomics, as this model provides the most comprehensively curated metabolite-to-pathway mapping for Proteobacteria [87]. Studies have demonstrated that *E. coli* is a reliable reference for other Gram-negative species due to the high degree of functional orthology in core primary metabolic pathways [88,89]. However, while this gold-standard reference effectively identifies the systemic shifts characterizing the VBNC state, it may not fully capture the unique ecological and metabolic specializations of *A. hydrophila* [89]. The active regulation of energy hubs and signaling metabolites strongly suggests that *A. hydrophila* entered a reversible VBNC state; however, a formal resuscitation assay was not performed in our study. Previous studies have established that foodborne pathogens like *E. coli* and *Salmonella enteritis* induced into dormancy by cold and osmotic stress can regain culturability upon the removal of stressors, such as an increase in temperature or the addition of specific nutrients [90,91]. The downregulation of growth-related metabolites observed in our results is consistent with the “stringent response” required for these cells to survive until conditions become favorable for resuscitation [92]. This integrated metabolic strategy enables *A. hydrophila* to persist in a dormant yet viable state, thereby posing continued risks in food and environmental reservoirs.

## 5. Conclusions

This study elucidates the metabolomic basis of the VBNC state in *A. hydrophila* under a simulated hurdle environment consisting of salinity and sodium benzoate and the survival mechanisms of pathogens subjected to specific preservation stressors commonly encountered in seafood processing. A panel of candidate metabolites was identified as putative biomarkers and reliably distinguished VBNC cells from culturable populations. VBNC induction was associated with suppression of growth- and signaling-related metabolites, including cyclic AMP, aminoadipic acid, hypotaurine, O6-CM-dG, and betaine, coupled with enrichment of stress-adaptive metabolites such as proline, pipecolic acid, urocanic acid, azelaic acid, and orcinol. Pathway analysis further revealed coordinated perturbations in amino acid metabolism and nucleotide metabolism. Importantly, the findings demonstrate that exposure to saline and sodium benzoate, widely regarded as bacteriostatic preservatives, can promote VBNC formation rather than complete microbial inactivation, thereby allowing pathogenic bacteria to persist in a metabolically altered but viable state. These insights have significant implications for food safety and public health, highlighting the need to reconsider preservation strategies and develop VBNC-targeted monitoring tools to mitigate hidden microbial risks in preserved foods.

## Figures and Tables

**Table 1:** Time-matched comparison of the literature-based selected metabolites at day 30, identified as potential biomarkers distinguishing the control and VBNC groups. The table provides the details of mean intensity, fold change, and significance of each metabolite.

Metabolite Name	Mean Intensity (Control)	Mean Intensity (VBNC)	log_2_FC	Significance at Day 30	*p* Value	References
Cyclic AMP	20,567.3	0	10.01	Downregulated	2.39 × 10^−9^	[34]
Aminoadipic acid	6465.3	0	8.34	Downregulated	1.82 × 10^−9^	[35,36,37,38]
Hypotaurine	46,568.7	0	11.19	Downregulated	5.59 × 10^−9^	[39]
O6-CM-dG	7169.33	0	8.49	Downregulated	1.78 × 10^−8^	[40]
Betaine	7861.33	186	4.5	Downregulated	0.0056	[41]
Urocanic acid	174.7	3237	5.1	Upregulated	0.0009	[42]
Pipecolic acid	252.6	9316.7	6.1	Upregulated	2.67 × 10^−7^	[35,36,37,38]
Proline	544	21,457	6.19	Upregulated	4.22 × 10^−7^	[43]
Azelaic acid	0	10,712	9.97	Upregulated	4.97 × 10^−8^	[44,45]
Orcinol	185	15,512	7.28	Upregulated	9.90 × 10^−5^	[44,45]

Note: log_2_FC (fold change) measures the relative abundance of metabolites in the VBNC group relative to the control group (VBNC/Control). Detailed statistical metrics, including *p*-values and FDR values for all identified metabolites, are provided in Supplementary Table S3.

## Data Availability

The original contributions presented in this study are included in the article/Supplementary Materials. Further inquiries can be directed to the corresponding author.

## Supplementary Materials

The following supporting information can be downloaded at: https://www.mdpi.com/article/10.3390/foods15081289/s1, Table S1: Total Plate Count (TPC) and viability assessment via flow cytometry. Data represents TPC, log CFU/mL, cell count, and cell percentage for control and treatment group; Table S2: Heat map analysis and ANOVA post-hoc comparison between the time intervals for control and treatment group; Table S3: Statistical values for volcano plot analysis showing differential metabolites and putative biomarkers in *A. hydrophila* for Control (Day 30/Day 0), Treatment (Day30/Day 0) and treatment/control (Day30/Day 30); Table S4: Statistical values for pathway analysis and metabolite category enrichment analysis; Figure S1: PERMANOVA analysis for control group; Figure S2: PERMANOVA analysis for treatment group; Figure S3: Heatmap of clustering hierarchical analysis for control group; Figure S4: Heatmap of clustering hierarchical analysis for treatment group.
