# Supplementary figures and images for: Untargeted Metabolomics Reveals Metabolic Reprogramming During Viable but Non-Culturable State Formation in Aeromonas hydrophila Under Preservative Stress
Source: Foods. 2026 Apr 9;15(8):1289. doi: 10.3390/foods15081289 (PMC13114279; doi:10.3390/foods15081289)

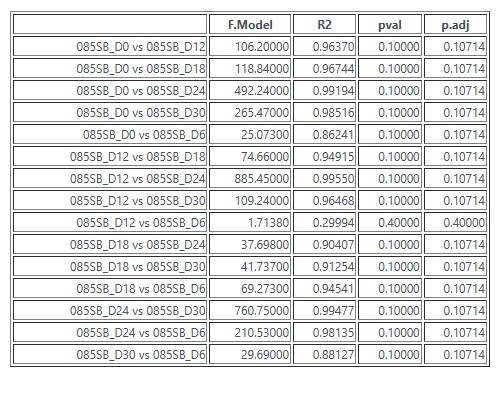

Supplement: Supplementary file 1 [file foods-15-01289-s001.zip › supplementary Figure S1.jpg]

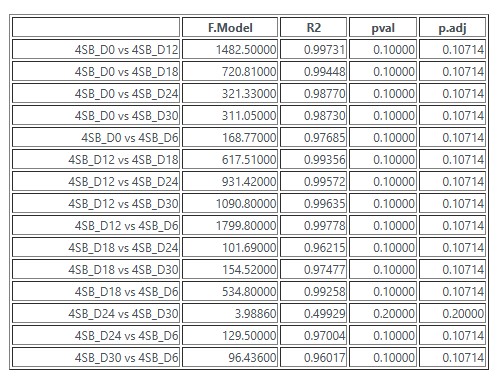

Supplement: Supplementary file 1 [file foods-15-01289-s001.zip › supplementary Figure S2.jpg]

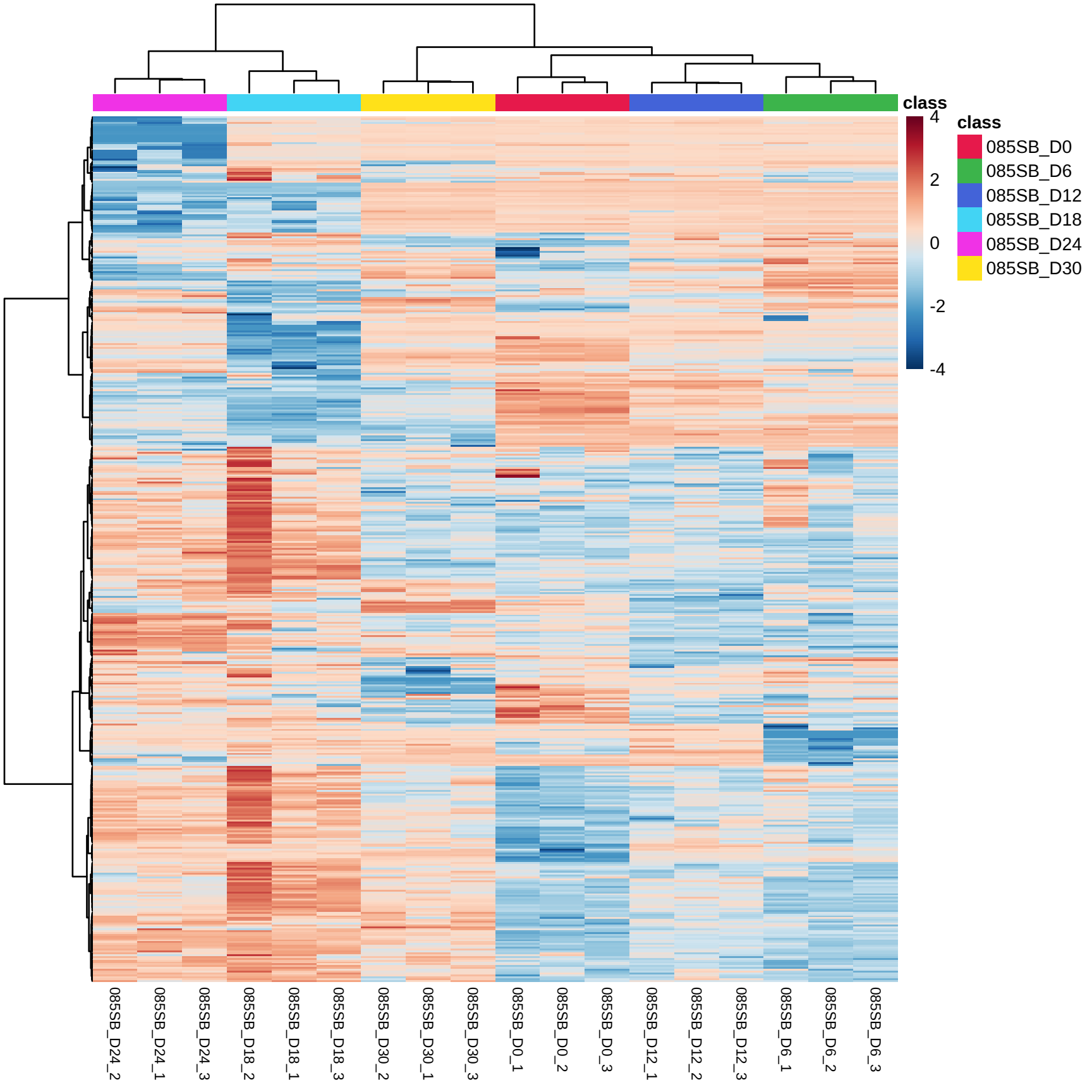

Supplement: Supplementary file 1 [file foods-15-01289-s001.zip › supplementary Figure S3.pdf]

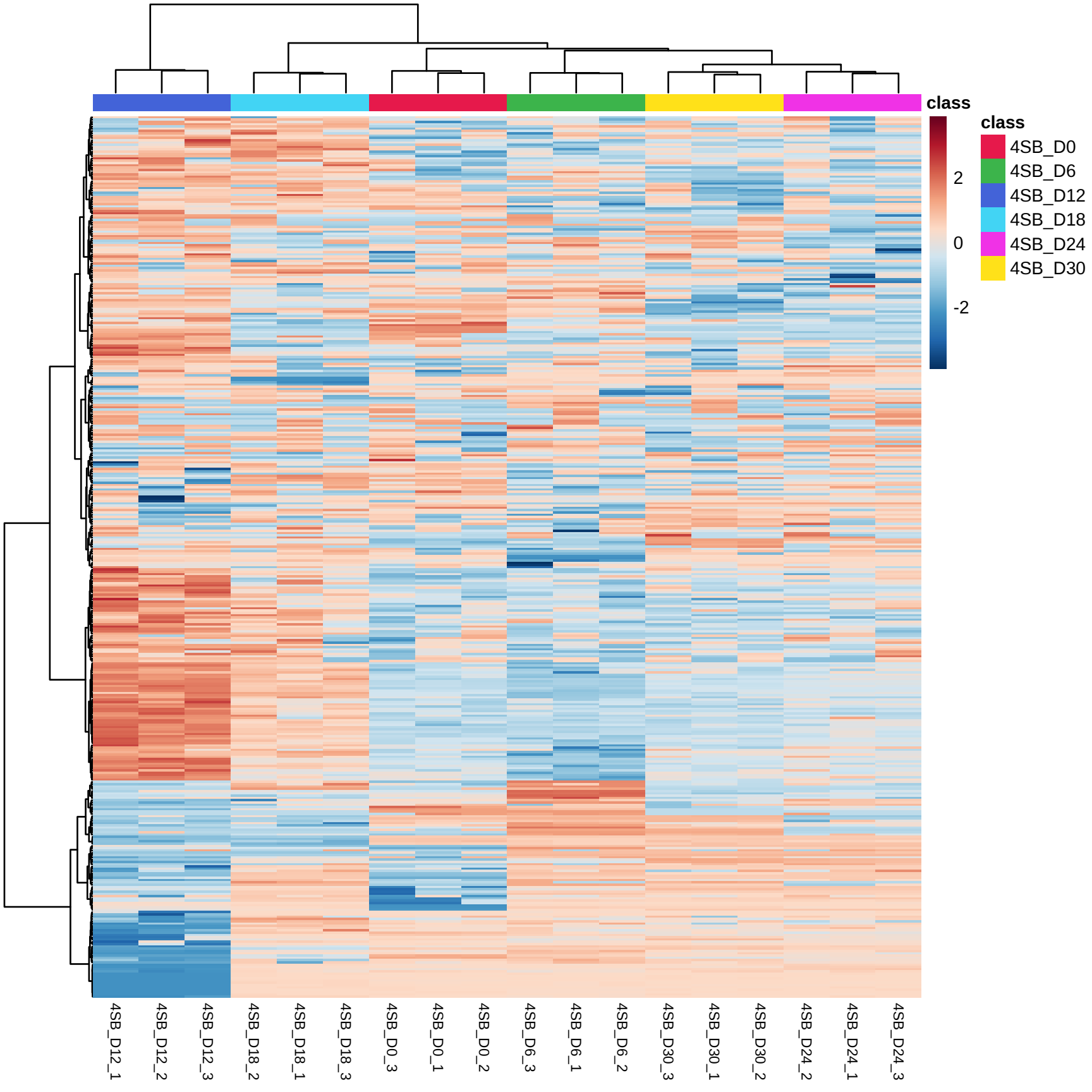

Supplement: Supplementary file 1 [file foods-15-01289-s001.zip › supplementary Figure S4.pdf]
